# EPOR_2_/βcR_2_-independendent effects of low-dose epoetin-*α* in porcine liver transplantation

**DOI:** 10.1042/BSR20171007

**Published:** 2017-12-05

**Authors:** Linus Kebschull, Leon Franz Christoph Theilmann, Annika Mohr, Wencke Uennigmann, Sandra Stoeppeler, Barbara Heitplatz, Hans-Ullrich Spiegel, Ralf Bahde, Daniel Michael Palmes, Felix Becker

**Affiliations:** 1 Department of General and Visceral, Division of Surgical Research Surgery, University Hospital Muenster, Muenster, Germany; 2 Gerhard Domagk Institute of Pathology, University Hospital Muenster, Muenster, Germany

**Keywords:** Erythropoietin, endothelial nitric oxide synthase, ischemia-reperfusion injury, liver transplantation

## Abstract

Ischemia–reperfusion injury (IRI) remains a key component of graft damage during transplantation. Erythropoietin (EPO) induces anti-inflammatory and anti-apoptotic effects via the EPOR_2_/βcR_2_ complex, with a potential risk of thrombosis. Previous work indicates that EPO has EPOR_2_/βcR_2_-independent protective effects via direct effects on the endothelium. As the EPOR_2_/βcR_2_ receptor has a very low affinity for EPO, we aimed to test the hypothesis that EPO doses below the level that stimulate this receptor elicit cytoprotective effects via endothelial stimulation in a porcine liver transplantation model. Landrace pigs underwent allogenic liver transplantation (follow-up: 6 h) with a portojugular shunt. Animals were divided into two groups: donor and recipient treatment with low-dose EPO (65 IU/kg) or vehicle, administered 6 h before cold perfusion and 30 min after warm reperfusion. Fourteen of 17 animals (82.4%) fulfilled the inclusion criteria. No differences were noted in operative values between the groups including hemoglobin, cold or warm ischemic time. EPO-treated animals showed a significantly lower histopathology score, reduced apoptosis, oxidative stress, and most important a significant up-regulation of endothelial nitric oxide (NO) synthase (eNOS). Donor and recipient treatment with low-dose EPO reduces the hepatic IRI via EPOR_2_/βcR_2_-independent cytoprotective mechanisms and represents a clinically applicable way to reduce IRI.

## Introduction

Ischemia–reperfusion injury (IRI) remains the most crucial factor in the multifaceted sequence of graft damage in solid organ transplantation. Ischemia-induced apoptosis and necrosis during cold storage and oxygen radical induced damage during warm reperfusion provoke an inflammatory response with deleterious effects on short- and long-term outcomes [1]. In order to account for the increase in organ shortage, more and more marginal organs are used [2]. However, pre-existing and especially IR-related marginal organ qualities are amongst the most important risk factors for impaired graft function and graft failure [3]. Therefore, strategies to reduce IRI in solid organ transplantation are desperately needed. Amongst the promising compounds to exhibit protective effects in the sequence of IRI is erythropoietin (EPO).

EPO (a member of the type 1 cytokine superfamily) and its derivatives are routinely used to induce erythropoiesis by stimulating bone marrow erythrocytic progenitors to transform into mature red blood cells [4]. This classic erythropoietic effect depends on binding of EPO to the transmembrane EPO receptor (EPOR, a member of the type I cytokine receptor family) causing the formation of a homodimeric receptor complex (EPOR_2_), with subsequent activation of the receptor-associated Janus-activated kinase 2 (JAK2) [5]. As a result, two main intracellular downstream signaling cascades are activated including signal transducer and activator of transcription 5 (STAT5) and phosphatidylinositide 3-kinase/AKT (PI3K/AKT).

In addition, EPO has recently been shown to exert local tissue protective anti-inflammatory and anti-apoptotic (cytoprotective) effects through an alternative pathway. Specifically, Brines et al. [6] described that EPOR and a common β receptor (βcR) form a heterodimeric receptor complex (EPOR_2_/βcR_2_), which mediates distinct cytoprotective effects [7]. As the main ligand, EPO exhibits different receptor affinities for both receptors, with a rather low receptor affinity for the erythrocytic EPOR_2_, resulting in high doses being necessary to stimulate the cytoprotective EPOR_2_/βcR_2_ complex [8]. Thus, when high-dose EPO regimes have been used in clinical studies in the setting of kidney transplantation, an elevated short-term (within the first month) as well as long-term (12 months) risk for thrombotic events have been described [9]. However, recent reports suggest novel direct mechanisms for EPO-induced endothelial activation. These mechanisms include an EPOR-independent heterodimeric receptor complex formed by βcR_2_ and the vascular endothelial growth factor receptor 2 (VEGFR-2) [10] or even pathways independent of the EPOR_2_/βcR_2_ complex [11].

EPO mediates an array of vital, non-erythropoietic, cytoprotective effects in various IRI models including the brain [12], heart [13], kidney [14], lung [15], and intestine [16]. In models of hepatic IRI, EPO-induced cytoprotective mechanisms involving a reduction in oxidative stress and caspase-3 activation [17], decreased c-Jun N-terminal kinase phosphorylation [18], reduced nuclear factor-κB expression [19] as well as up-regulation of heme oxygenase-1 [20], all of which offer protection against IRI in terms of reduction in cellular apoptosis and necrosis, improved angiogenic recovery [18], as well as improvement in liver function and hepatic cellular integrity. In summary, EPO prevents ischemic cell death and reduces the development of secondary, proinflammatory cytokine-induced injury during reperfusion.

Amongst the best-characterized endothelial downstream targets of EPO in the IRI sequence is the phosphorylation and thus enhanced activation of endothelial nitric oxide (NO) synthase (eNOS). EPO-mediated phosphorylation of eNOS is facilitated by the EPOR mediated JAK2/PI3/AKT pathway, the βcR-dependent activation of AMP-activated protein kinase (AMPK) or activation of the βcR_2_–VEGFR-2 complex with currently unknown downstream signals [21]. While up-regulation of eNOS has been shown to be a crucial protective factor in renal IRI [21] and eNOS has been demonstrated to be a direct endothelial target of EPO [10], there are, however, currently no reports linking the cytoprotective effects of EPO in hepatic IRI to a direct endothelial activation, specifically to eNOS up-regulation.

Therefore, encouraged by the substantial body of evidence demonstrating cytoprotective effect of EPO in IRI models and mounting evidence of direct EPO-mediated effects on the endothelium, we aimed to test the hypothesis that low-dose (65 IU/kg b.w.) EPO elicits direct endothelial stimulation by up-regulation of eNOS. Thus, the present study examines the effect of low (concerning the EPOR_2_/βcR_2_–receptor complex, potentially subtherapeutic) doses of EPO in the sequence of hepatic IRI in a clinically relevant porcine liver transplant model with special focus on endothelial activation.

## Methods

### Animals

Principles of Laboratory Animal Care (NIH Publication volume 25, number 28, revised 1996) were followed and all procedures were conducted in accordance with the German Animal Welfare Law and approved by the local animal care committee (permit number: 8.87-50.10.36.09.021). Thirty-four female landrace pigs (weighing 40–45 kg, 3–6 months of age) were used as either donor or recipient animal for a total of 17 allogenic liver transplantations. Animals were kept in the central animal facility of the University of Muenster with free access to water and standard chow. All experiments were performed during the same daytime to prevent the previously described circadian influence on EPO-mediated effects [22,23].

### Groups

Animals were randomly assigned into two groups: donor and recipient treatment with 65 IU/kg body weight of the recombinant human EPO epoetin-α (EPO, Abseamed, Medice, Iserlohn, Germany) or saline vehicle (SHAM) treatment. EPO and saline vehicle were administrated intravenously (i.v.) 6 h before cold perfusion (donor preconditioning) as well as 30 min after reperfusion (recipient treatment). Seven animals per group were regarded as sufficient for statistical analysis.

### Anesthesia and monitoring

After fasting for 12 h, animals were sedated by intramuscular (i.m.) injection of azaperon (3 mg/kg b.w.) and ketamine (15 mg/kg b.w.). After first venous access was established by cannulation of an ear vein, anesthesia was completed by endotracheal intubation under continuous i.v. application of ketamine (10 mg/kg b.w.) and ethomidate (1.25 mg/kg b.w.). Maintenance of anesthesia was effectuated by using nitrous oxide and oxygen (2:1) with isoflurane (1.5 vol%). Additionally, a fentanyl bolus was given i.v. all 4–6 h (0.2 mg/kg b.w.). During the complete experiment, animals were kept under continuous anesthesia with constant monitoring of pulse, blood pressure, oxygen, and carbon dioxide levels as well as body temperature and intermittent blood gas analysis.

### Donor operation

Median laparotomy was followed by complete mobilization of the liver, suturing of all three diaphragmatic veins, and dissection of the hepatoduodenal ligament. The hepatic artery was dissected down to the celiac trunk and the gastroduodenal artery as well as the left gastric artery stump was preserved. The portal vein was isolated and the bile duct identified. The infrarenal aorta was cannulated, the animal then fully heparinized (25000 IU heparin) and after clamping of the supradiaphragmatic aorta, the abdominal aorta was flushed with 5 l of ice-cold HTK-Solution (Custodiol, Köhler Chemie, Alsbach-Hähnlein, Germany) at 120 mmHg. The vena cava was vented immediately by opening the supradiaphragmatic vena cava. Topical cooling was performed with crushed frozen saline and ice-cold saline rinse. The liver graft was then removed and back table preparation included further perfusion with HTK-Solution (hepatic artery with 0.25 l at 120 mmHg and portal vein with 0.75 l at 10–15 mmHg) followed by removal of the gall bladder after which the liver graft was stored in ice-cold HTK-Solution until implantation.

### Recipient operation

**Figure 1:**
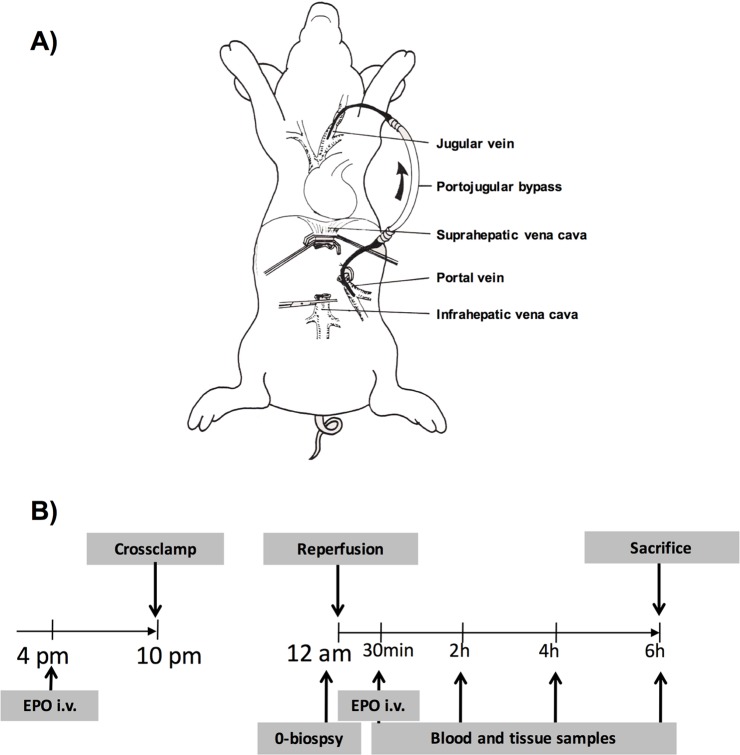
Schematic overview of the recipient operation with a portojugular bypass and the experimental design (**A**) Pigs underwent allogenic liver transplantation with a portojugular bypass between the right jugular vein and portal vein. Following established bypass flow, the liver graft was implanted by first anastomosing the suprahepatic and infrahepatic vena cava. Then the bypass was clamped and anastomosis of the portal vein was conducted followed by portal reperfusion and last, the arterial anastomosis was performed. The bile duct was cannulated for collection of bile. (**B**) Animals were randomly assigned to two groups: donor and recipient treatment with 65 IU/kg body weight of the recombinant human EPO epoetin-α (EPO) or saline vehicle (SHAM) treatment. EPO and saline vehicle were administrated i.v. for 6 h before cold perfusion (donor preconditioning) as well as 30 min after reperfusion (recipient treatment). All experiments were performed during the same daytime to prevent the previously described circadian influence on EPO-mediated effects.

The recipient animal was prepared as described above. On the right external carotid artery, an arterial catheter was inserted for continuous blood pressure management, a central venous line and a rapid catheter were inserted into the right jugular vein. To account for the risk of serious cardiovascular complications during total portal vein obstruction in the anhepatic phase, we used a model with a portojugular shunt [24,25]. The cervical left hand side was prepared for insertion of a heparin-coated silicone tube for a portojugular bypass. Following laparotomy, the liver was mobilized and the hilum was dissected close to the liver (Figure 1).

Bile duct and artery were transected and the portal vein prepared. After insertion of the flushed bypass tube into the jugular vein, the portal vein was clamped and cut in the hepatic hilum. The bypass was inserted and fixed using a Prolene 4/0 purse string suture and an additional ligature. The donor liver was flushed with an additional l HTK-Solution on the back table and implanted by first anastomosing suprahepatic and infrahepatic vena cava (4/0 Prolene), then reopening of the vena cava to prevent kidney injury and to maintain hemodynamic stability. After clamping of the bypass and anastomosis of the portal vein (5/0 Prolene), the liver was portally reperfused and the arterial anastomosis was performed (7/0 Prolene). The bile duct was cannulated for collection of bile. For further standardization, all recipient operations were performed by the same surgeon (L.K.).

### Perioperative management

Following intubation, volume was continuously resuscitated with sterofundine and HAES solution at a rate of 7.5 ml/h. During the anhepatic phase, volume replacement was augemented and if necessary, arterenol was administered to keep blood pressure and heart rate constant. Criteria for a surgically successful operation were survival until study end point, hemoglobin >7 mg/dl. At the end of each experiment, animals were killed by a lethal dose of T61®.

### Sample collection

For all animals, a standardized sample collection procedure was established: 30 min, 2, 4, and 6 h after reperfusion blood samples and incisional liver biopsies were taken in standardized fashion from the left liver lobe. Between the samples, the abdomen was closed and the animal was positioned in the physiological belly-down position. Tissue samples were either formalin-fixed or snap-frozen in liquid nitrogen and subsequently stored at −80°C. Serum samples were analyzed for aspartate aminotransferase (AST), alanine aminotransferase (ALT), lactate dehydrogenase (LDH), glutamate dehydrogenase (GLDH), γ-glutamyl transpeptidase (γ-GT), and creatinine at the Central Laboratory of the University Hospital Muenster, according to human blood protocols.

### Histology

Tissue samples were fixed in formalin, embedded in paraffin, and cut into 4-μm sections, which were subsequently stained with Hematoxylin–Eosin (H&E) and Azan. Histologic examination was performed by a board-certified pathologist (B.H.) blinded to the experimental layout. To quantitate liver damage following IRI, a modified Suzuki Score [26] was used. This score classically includes (I) sinusoidal congestion, (II) vacuolization as well as (III) hepatocyte necrosis and was modified by adding (IV) sinusoidal inflammation to account for the post IRI inflammatory cell infiltration. Each parameter was scored from 0 to 4 (0 = none, 1 = minimal, 2 = mild, 3 = moderate, 4 = severe for I, II, IV and 0 = none, 1 = single cell necrosis, 2 = up to 30% necrosis, 3 = up to 60% necrosis, 4 = >60% necrosis for III) based on the extent of injury at each time point after reperfusion, subsequently added and thus an average score calculated for each group.

### Immunohistochemistry

For immunohistochemistry analysis, liver tissue was collected 6 h after reperfusion, sections were obtained as described above and stained for markers of endothelial activation (eNOS), liver cell apoptosis (ssDNA) [27–29] and the established marker of oxidative nuclear stress, 8-hydroxydeoxyguanosine (8-OHG, [30]). eNOS was detected using a polyclonal rabbit antibody (Transduction Laboratories, Lexington, KY, U.S.A.) and apoptotic cells were detected with a rabbit primary antibody against ssDNA (IBL Co. Ltd., Gunma, Japan) using the DAKO anti-rabbit En-Vision-HRP (DAKOcytomation) and NovaRed substrate kit (Vector Laboratories, Burlingame, CA, U.S.A.) with H&E counterstaining. For the analysis, 20 fields (at ×200 magnification) were randomly chosen and reviewed and scored by two independent investigators blinded to the experimental layout using standardized scoring system, previously established for endothelial activation in hepatic IRI [31]. Accordingly, the extent of positive stained cells was scored a four-grade scale with (0) negative; (1) partially (0–25%) weak positivestained cells; (2) partially (25–50%) moderate or diffused weak positive cells; (3) diffuse moderate or strong positive cells (50–75%), and (4) diffuse strong positive cells (>75%). Nuclear oxidative stress was detected with a monoclonal mouse primary antibody against 8-OHG (Abcam, Cambridge, U.K.) using the DAKO goat anti-mouse En-Vision-HRP and ACE substrate chromogen kit (DAKOcytomation) with Hematoxylin counterstaining. For the analysis of oxidative stress, three high-power fields (HPF, at ×400 magnification) were randomly chosen, reviewed and scored (number of 8-OHG-positive cells) by two independent investigators blinded to the experimental layout. Results were expressed as 8-OHG-positive cells per HPF.

### Statistical analysis

All statistical analyses were performed using GraphPad Prism 6 (GraphPad Software, La Jolla, CA, U.S.A.). Groups were compared with a two-tailed, unpaired Student’s *t* test, or one- or two-way ANOVA followed by Bonferroni’s post hoc testing, as appropriate. Data were expressed as mean ± S.E.M. and differences were considered statistically significant when *P*<0.05.

## Results

### Survival and inclusion

Fourteen of 17 (82.4%) transplanted pigs fulfilled the inclusion criteria for a surgically successful operation in terms of survival until study end point and hemoglobin >7 mg/dl. Three animals were excluded from further analysis based on severe hemorrhage and premature death.

### Perioperative values

When comparing initial preoperative values before allogenic liver transplantation (see Table 1), no significant differences between the groups (SHAM compared with EPO) were found in terms of donor body weight (SHAM: 40.6 ± 0.8 kg compared with EPO: 41.1 ± 1.8 kg), recipient body weight (SHAM: 42.7 ± 2.2 kg compared with EPO: 43.3 ± 1.7 kg), recipient Hb (SHAM: 8.9 ± 0.1 mg/dl compared with EPO: 9.2 ± 0.3 mg/dl). We observed a marginal, but significant (*P*=0.03) difference between cold ischemia time (SHAM: 13.5 ± 0.06 h compared with EPO: 13.9 ± 0.1 h) as well as a trend (*P*=0.08) toward a longer warm ischemia time in EPO-treated pigs (SHAM: 46.4 ± 0.7 compared with EPO: 44.10 ± 1.0 min). Thus, based on these results, we can exclude any perioperative or organ storage based bias.

**Table 1:** Pre- and intraoperative values in allogenic porcine liver transplantation

	Sham (*n*=7)	EPO (*n*=7)	
Donor body weight (kg)	40.6 ± 0.8	41.1 ± 1.8	*P*=0.8
Recipient body weight (kg)	42.7 ± 2.2	43.3 ± 1.7	*P*=0.8
Recipient Hb (mg/dl)	8.9 ± 0.1	9.2 ± 0.3	*P*=0.5
Cold ischemia (h)	13.5 ± 0.06	13.9 ± 0.1	*P*=0.03
Warm ischemia (min)	46.4 ± 0.7	44 ± 1	*P*=0.08

SHAM and EPO-treated animals were compared in terms of donor body weight, recipient body weight, recipient Hb, or cold and warm ischemia time.

### Serum chemistry

**Figure 2:**
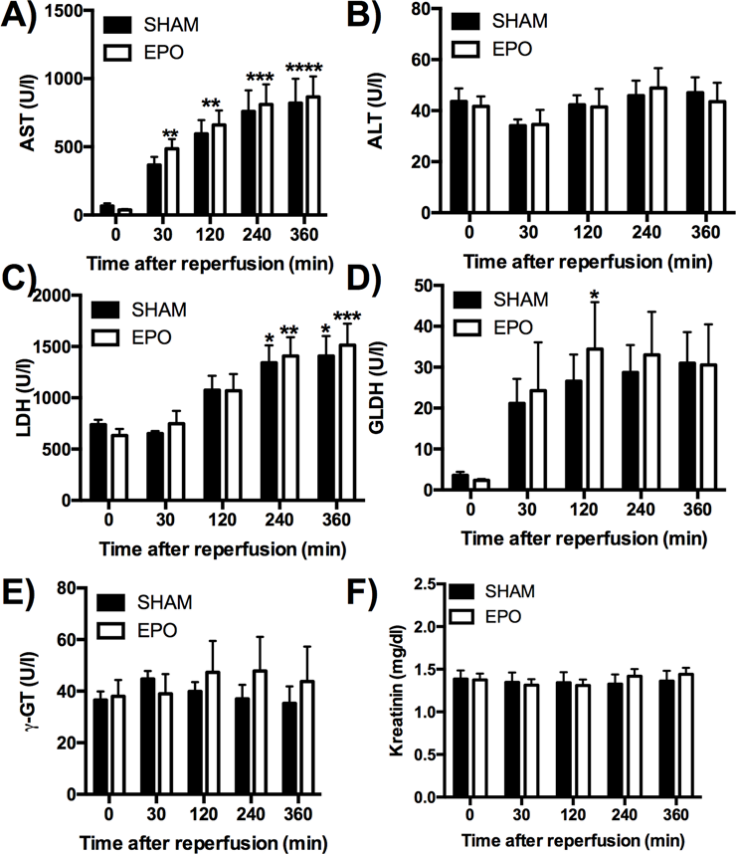
Effects of low-dose recombinant human EPO epoetin-α (EPO) on serum liver enzymes 6 h after reperfusion Serum markers for hepatic: (**A**) AST, (**B**) ALT, (**C**) LDH, (**D**) GLDH, (**E**) γ-GT as well kidney function (**F**) creatinine were analyzed over the 6-h course of reperfusion at 0, 30, 120, 240, and 360 min. Data are expressed as mean ± S.E.M.; *n*=5–7 pigs/group. *P<0.05, **P<0.01, ***P<0.001, ****P<0.0001 compared with respective control baseline counterpart.

We next analyzed serum markers for hepatic as well kidney function over the 6-h time course of reperfusion in our model of allogenic liver transplantation. Animals in both groups (SHAM and EPO) showed a steady and significant increase in serum AST levels with a peak at 360 min after reperfusion (SHAM: 820.6 ± 177.6 U/l and EPO: 865.8 ± 149.4 U/l), while no significant differences were noted when comparing SHAM with EPO animals (Figure 2A). When analyzing serum ALT levels, no significant differences were found neither within nor between experimental groups (Figure 2B). LDH, typically detected at higher levels in pigs, showed a significant rise in both groups as early as 240 min after reperfusion (SHAM: 1342 ± 169.3 U/l and EPO: 1407 ± 183.9 U/l, Figure 2C), while no significant differences were found between SHAM and EPO-treated animals. GLDH peaked in EPO-treated animals at 120 min after reperfusion (34.43 ± 11.47 U/l) while no significant elevation was noted in SHAM animals (Figure 2D). γ-GT (Figure 2E) as well as creatinine (Figure 2F) showed no significant changes in both groups and remained stable throughout the study.

### Histological damage

**Figure 3:**
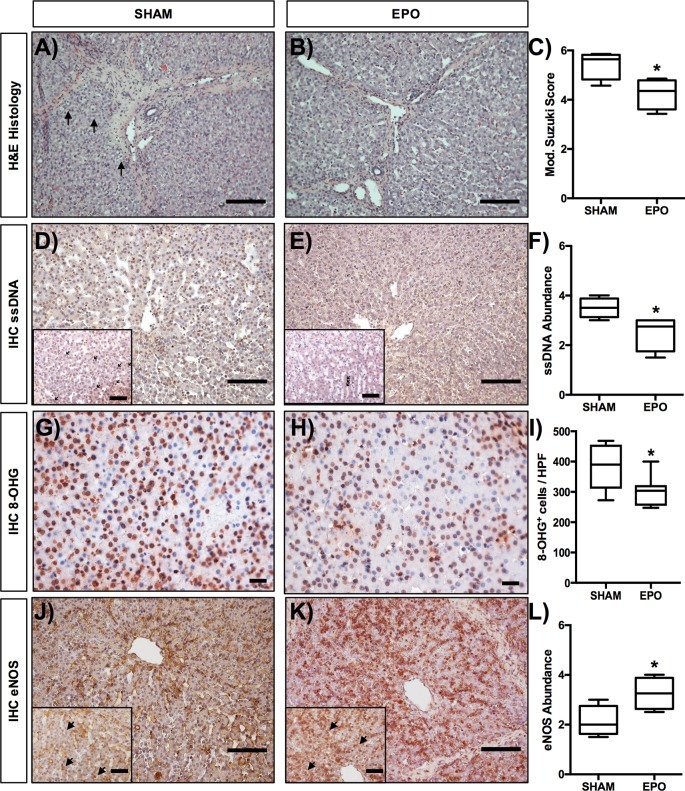
Effects of low-dose recombinant human EPO epoetin-α (EPO) on morphology, apoptosis, and eNOS expression Histological assessment for hepatic injury in H&E-stained sections following allogenic liver transplantation in (**A**) SHAM and (**B**) EPO-treated animals was based on (**C**) modified Suzuki criteria, including (I) sinusoidal congestion, (II) vacuolization, (III) hepatocyte necrosis, and (IV) sinusoidal inflammation. Each parameter was scored from 0 to 4 (0 = none, 1 = minimal, 2 = mild, 3 = moderate, 4 = severe for I, II, IV and 0 = none, 1 = single cell necrosis, 2 = up to 30% necrosis, 3 = up to 60% necrosis, 4 = >60% necrosis for III) based on the extent of injury at each time point after reperfusion, subsequently added and thus an average score calculated for each group. Liver cell apoptosis (ssDNA) was analyzed by immunohistochemistry in (**D**) SHAM as well (**E**) EPO-treated animals and (**F**) scored by grading the extent of positive stained cells on a four-grade scale with (0) negative; (1) partially weak positive stained cells (0–25%); (2) partially moderate or diffuse weak positive cells (25–50%); (3) diffuse moderate or strong positive cells (50–75%) and (4) diffuse strong positive cells (>75%) grade. Oxidative injury was analyzed by immunohistochemistry using the established marker of oxidative nuclear stress, 8-OHG. For the analysis of oxidative stress, three HPF (at ×400 magnification) were randomly chosen from SHAM (**G**) and EPO-treated animals (**H**), reviewed and scored (number of 8-OHG-positive cells) by two independent investigators blinded to the experimental layout. Results were expressed as 8-OHG-positive cells per HPF (**I**). The expression of eNOS was also analyzed by immunohistochemistry (IHC) in (**J**) SHAM as well (**K**) EPO-treated animals and (**L**) analyzed accordingly. Overview images were taken at ×200 magnification, insets (and 8-OHG images) at ×400 magnification and 20 randomly chosen fields (at ×200 magnification) were analyzed per slide; scale bar =50 μm. Data are expressed as mean ± S.E.M.; *n*=4–7 pigs/group. *P<0.05 compared with SHAM.

Hepatic sections were scored with an extent-based score from 0 to 4 for modified Suzuki criteria [26], including (I) sinusoidal congestion, (II) vacuolization as well as (III) hepatocyte necrosis, and (IV) sinusoidal inflammation. Figure 3 shows H&E stained hepatic sections from SHAM and EPO-treated animals and displays the reduced vacuolization in EPO-treated animals following liver transplantation (Figure 3A,B, respectively). Histological assessment using modified Suzuki criteria (Figure 3C) revealed a significant (*P*=0.0122) decrease in the severity and extent of IRI in EPO treated (4.25 ± 0.3) animals compared with SHAM animals (5.43 ± 0.26).

### Immunohistochemistry

Since it is known that anti-apoptotic and antioxidative effects are amongst the key mechanisms for cytoprotective effects of EPO, we assessed liver cell apoptosis and oxidative injury following allogenic liver transplantation by staining for ssDNA and 8-OHG. The appearance of ssDNA or 8-OHG positive stained cells was scored in liver sections from SHAM (Figure 3D,G) and EPO-treated (Figure 3E,H) animals obtained 6 h after reperfusion, using an extend-based score. We found the abundance of ssDNA to be significantly (*P*<0.05, Figure 3F) lower in EPO-treated animals (2.5 ± 0.2) than in SHAM animals (3.5 ± 0.35) demonstrating reduced hepatic apoptosis. In addition, we found a significantly lower number (*P*<0.05. Figure 3I) of 8-OHG stained cells in EPO-treated animals (304.9 ± 19.14 cells per HPF) compared with SHAM animals (380.3 ± 27.13 cells per HPF), suggesting a reduction in oxidative stress induced by EPO treatment.

Since it is further known that EPO can activate non-erythropoietic but rather cytoprotective pathways, via receptors other than the EPOR_2_/βcR_2_ complex, we next thought to evaluate pathways by which EPO might directly act on the endothelium, by assessing the up-regulation of the key endothelial enzyme eNOS, which has been suggested to be a direct target of EPO on the endothelium. When we stained liver sections from SHAM (Figure 3J) and EPO-treated (Figure 3K) animals obtained 6 h after reperfusion in the IRI sequence following allogenic liver transplantation, we found a significant (*P*<0.05, Figure 3L) increase in cells stained positive for eNOS in EPO-treated animals (3.25 ± 0.32) compared with SHAM animals (2.13 ± 0.31), suggesting an up-regulation of this important endothelial enzyme.

## Discussion

There are four novel principle findings in the present study: (i) low-dose EPO donor preconditioning and recipient treatment in a porcine liver transplant model results in lower tissue damage 6 h after reperfusion. (ii) This improvement is accompanied by a reduction in liver cell apoptosis as well as (iii) a reduction in oxidative injury, and (iv) there is a higher hepatic eNOS expression 6 h after reperfusion. In addition, it is important to note two very specific translatable key aspects of our study: (i) we used a well standardized and translational porcine liver transplant model with strict inclusion criteria and (ii) the reported warm and cold ischemic times are comparable with the clinical setting in humans, since cold ischemic times of 13.5 ± 0.06 and 13.9 ± 0.1 h are found often in marginal grafts [32] that could profit from a peri- and postoperative treatment augmenting graft quality.

EPO eliciting an array of vital non-erythropoietic cytoprotective effects by preventing ischemic cell death and reducing the development of secondary, proinflammatory cytokine-induced injury during reperfusion in various IRI models, including the liver [17–20]. However, these cytoprotective effects are axiomatically linked to EPO-induced erythropoietic mechanisms of promoting proliferation and differentiation, as well as preventing apoptosis in erythocytic progenitors and hematopoietic effects [8]. Thus, possible side effects of EPO treatment using doses directed for cytoprotection (500–5000 IU/kg b.w.) include an acceleration of megakaryocyte maturation [33] leading to the release of highly reactive platelets [34] and EPO-mediated enhancement of endothelial cell activation as well as increased expression of adhesion molecules [35]. These EPO-associated hematopoietic effects further enhance leukocyte–platelet interactions and stimulation of vascular smooth muscle contraction, which diminishes regional blood flow. All these contributing factors enhance thrombus formation, which can lead to heightened mortality and morbidity. This has become evident in recent clinical trials which have reported an increase in thrombosis, affiliated with the endogenous high dose (total dose of 100.000 IU over 3 consecutive days) and therefore potentially cytoprotective effect of high dose EPO treatment [7]. This dichotomous (both erythropoietic and cytoprotective protective) role is based on EPO interactions with different receptors, namely the cytoprotective EPOR_2_/ βcR_2_ complex and the erythropoietic EPOR_2_, with the later having a much lower EPO-binding affinity (1–10 pmol/l) than the first (2–20 nmol/l) [8].

To shed further light on this dilemma and to potentially overcome the problem of thrombotic side effects when using cytoprotective EPO doses, experimental studies examined whether the EPOR_2_/βcR_2_ complex is essential for the EPO-mediated cytoprotective effects. In transgenic knockout mice with EPOR_2_ being expressed only in bone marrow and on vascular endothelial cells, EPO was still protective in a model of traumatic brain injury as well as in cardiac ischemia [36,37]. The concept of an endothelial-specific EPO pathway was further supported by findings that when eNOS knockout mice were used in cardiac ischemia model, reduced protective effects of EPO were observed [37]. As for the βcR_2_, some *in vitro* studies proposed that the βcR_2_ receptor is essential for cytoprotective effects of EPO and accordingly EPO did not prove to be protective in βcR_2_ knockout mice after spinal cord injury [6]. Conversely, EPO derivatives were still protective after cardiac injury in βcR_2_ knockout mice [11], indicating alternative pathway(s) to be responsible for the protective effects of low-dose EPO on the endothelium. Recently, Sautina et al. [10] found that EPOR_2_ interacts with VEGFR-2 to elicit downstream signals in the endothelium and endothelial progenitor cells. These findings were further corroborated by Kanellakis et al. [11], who described EPO-mediated cytoprotective effects that were independent of the EPOR_2_/βcR_2_ receptor complex. Therefore, we used a novel approach by administering low-dose (65 IU/kg b.w.) EPO (concerning the EPOR_2_/βcR_2_ complex, potentially subtherapeutic) to avoid systemic thrombotic side effects but to achieve a local effect on the endothelium. Our findings of a reduced morphological hepatic damage and apoptosis and oxidative stress coupled with the potential mechanistic evidence of eNOS activation further support our hypothesis that low-dose EPO-induced effects can be transmitted by pathways other than the EPOR_2_/βcR_2_ pathway [8]. These EPOR_2_/βcR_2_-independent pathways seem to be favorable in transplant medicine, since low-dose EPO is used frequently for anemia treatment in clinical medicine and has not been linked to severe adverse events such as thrombosis [38]. Here we used a dose normally used for induction of erythropoesis in humans [39] and as such it is highly unlikely that there is a high rate of EPO-induced thrombosis in our study given the evidence stated above and the fact that no animal presented any sign of thrombosis.

While we failed to detect protective effects of low-dose EPO on serum levels of liver enzymes, we found clear evidence for reduced morphological hepatic damage as evidenced by the reduced modified Suzuki Score as well as the reduction in apoptosis (as quantitated by a lower ssDNA expression in EPO-treated livers). Since apoptosis as well as oxidative stress are amongst the main mechanisms involved in IRI, we aimed to investigate the influence of low-dose EPO donor preconditioning and recipient treatment on these critical cellular pathways. We found a significant reduction in apoptosis (as quantitated by a lower ssDNA expression in EPO-treated livers). The anti-apoptotic effects of EPO observed in this clinically relevant porcine *in vivo* study are in line with previous reports and presumably mediated via caspase-3 activation [17,40]. In addition, we found a significant decrease in nuclear oxidative stress (as quantitated by a lower 8-OHG expression in EPO-treated livers). These results confirm the findings by Sepodes et al. [17] of a decrease in oxidative hepatic injury following EPO treatment and suggest that low-dose EPO donor preconditioning and recipient treatment could offer protection to liver cells against a hallmark feature of IRI, which is cellular injury caused by reactive oxygen species [1]. However, more importantly, the main focus of our study was to detect direct EPO-mediated endothelial activation. Compelling evidence suggests eNOS to be a key target of EPO-induced endothelial activation. eNOS synthesizes NO from arginine and oxygen, which is a central humoral factor causing vascular smooth muscle relaxation through cGMP-mediated signal transduction pathways. Thus, eNOS is a crucial modulator responsible for vascular tone and blood flow, maintaining hepatic vascular homeostasis [41]. Specifically, eNOS-derived NO protects against liver IRI [42]. In *in vitro* studies, EPO has been shown to have direct effects several types of human endothelial cells and increased the bioavailability of NO by up-regulation of eNOS. In addition, effects of EPO on eNOS induction were absent under normoxia but were significantly increased under hypoxic conditions [43] which is in-line with the concept of an effect of low-dose EPO on eNOS expression in the IRI sequence. This further supports the idea of a donor pretreatment to deliver EPO before the ischemic insult. In our study presented here, we found that low-dose EPO treatment significantly increased hepatic eNOS expression in our clinically relevant *in vivo* model of auxiliary porcine liver transplantation. Several import factors should be considered when interpreting the eNOS finding. Besides the direct cytoprotective effects of EPO on apoptosis, eNOS up-regulation can increase vasodilatation, thus permitting sufficient hepatic perfusion in the IRI setting [44]. In addition to direct effect on blood flow, up-regulation of eNOS could also induce a diminished inflammatory response, since immune cell extravasation was increased in the lung in eNOS-deficient mice following hypoxic lung injury [45]. Due to the low receptor affinity of EPO to the EPOR_2_/βcR_2_ receptor and the unlikely activation of this pathway, we can conclude that the significant eNOS induction in the present *in vivo* study is possibly a result of direct endothelial activation.

While our novel findings suggest low-dose EPO to be cytoprotective in a clinical relevant and translatable porcine liver transplant model, we do recognize that our study has several limitations: (i) it was not possible to prove any effect on hematocrit due to short observation time after reperfusion coupled with the obligatory blood loss connected to the liver transplantation. (ii) A longer observation period of several weeks after transplantation could give more information concerning hematocrit development and adverse events as an elevated level of thrombosis. (iii) Long-term experiments could possibly detect long-term effects of perioperative EPO treatment on graft function and recovery in a liver transplant setting. These long-term experiments have the potential to close the gap in knowledge as to whether the observed improvement in histological damage, reduced apoptosis, and ameliorated oxidative injury might have further implication for human liver transplant recipients. (ii) In addition we used a model of donor preconditioning and recipient treatment and as such we cannot discriminate between the effects of EPO as a pharmacological preconditioning agent to prevent ischemic damage compared with the protective effects of EPO on the reperfusion injury. (v) Also, one could argue that a porcine model of allogenic liver transplantation without portojugular shunt, as published by Heuer et al., would be more translational since this procedure is not utilized in the majority of the liver transplantations in humans [48]. While we have previously tried to establish this model (results not shown), we have chosen to use a model with portojugular shunt to avoid the non-physiological venous congestion in the small bowel. (vi) While we provide novel data on the effects of low-dose EPO donor preconditioning and recipient treatment induced increase in eNOS expression in hepatic IRI, additional work is needed to further dissect this pathway. Follow-up research from the present study should include a more sophisticated analysis of the regulation of EPO-induced eNOS expression (on both the DNA and protein level) as well as IRI models including genetically modified animals. (vii) Follow-up research is needed to advance the understanding of the presented results of reduced apoptosis, which would include a more in depth analysis of molecular pathways including caspase-3 activity as well as the influence of oncogene-derived proteins such as Bax/Bcl-2 and the role of p53. In conclusion, further studies are needed to address these complex issues and are beyond the scope of this manuscript. In addition, current research attempts to reduce prothrombotic side effects by using EPO as an additive to preservation solutions [46], direct injection into the portal vein [40], and most promising, EPO derivatives without any erythropoietic effects [47].

To our knowledge, to date there are only two studies analyzing effects of EPO in models of liver transplantation. However, our study is the first large animal study examining EPO in a porcine liver transplant setting as well as the first to report EPO-mediated hepatic eNOS up-regulation in this setting. Our data prove that donor preconditioning and recipient treatment with low-dose EPO has imminent effects on histological graft damage and apoptosis rate, oxidative stress, and also induces eNOS after transplantation. We were able to show that low-dose EPO possibly reduces the IRI via pathways independent of the EPOR_2_/βcR_2_ receptor complex. In conclusion, donor preconditioning and recipient treatment with low-dose EPO represents an effective and clinically applicable way to ameliorate IRI in a transplant setting.

## Abbreviations

AST: aspartate aminotransferase
ALT: alanine aminotransferase
eNOS: endothelial nitric oxide synthase
EPO: erythropoietin
EPOR: EPO receptor
GLDH: glutamate dehydrogenase
HAES: hydroxyethyl starch
HPF: high-power field
HTK: histidine-tryptophan-ketoglutarate solution
H&E: hematoxylin–eosin
IRI: ischemia–reperfusion injury
i.v.: intravenously
JAK2: Janus-activated kinase 2
LDH: lactate dehydrogenase
VEGFR-2: vascular endothelial growth factor receptor 2
8-OHG: 8-hydroxydeoxyguanosine
βcR: β common receptor
γ-GT: γ-glutamyl transpeptidase
